# SLU7: A New Hub of Gene Expression Regulation—From Epigenetics to Protein Stability in Health and Disease

**DOI:** 10.3390/ijms232113411

**Published:** 2022-11-02

**Authors:** María Gárate-Rascón, Miriam Recalde, Carla Rojo, Maite G. Fernández-Barrena, Matías A. Ávila, María Arechederra, Carmen Berasain

**Affiliations:** 1 Program of Hepatology, Center for Applied Medical Research (CIMA), University of Navarra, Avda. Pio XII, n55, 31008 Pamplona, Spain; 2 IdiSNA, Navarra Institute for Health Research, 31008 Pamplona, Spain; 3 National Institute for the Study of Liver and Gastrointestinal Diseases (CIBERehd, Carlos III Health Institute), 28029 Madrid, Spain

**Keywords:** alternative splicing, gene expression, transcription, epigenetics, differentiation, stress, genomic stability, cell cycle, liver pathophysiology, cancer

## Abstract

SLU7 (Splicing factor synergistic lethal with U5 snRNA 7) was first identified as a splicing factor necessary for the correct selection of 3′ splice sites, strongly impacting on the diversity of gene transcripts in a cell. More recent studies have uncovered new and non-redundant roles of SLU7 as an integrative hub of different levels of gene expression regulation, including epigenetic DNA remodeling, modulation of transcription and protein stability. Here we review those findings, the multiple factors and mechanisms implicated as well as the cellular functions affected. For instance, SLU7 is essential to secure liver differentiation, genome integrity acting at different levels and a correct cell cycle progression. Accordingly, the aberrant expression of SLU7 could be associated with human diseases including cancer, although strikingly, it is an essential survival factor for cancer cells. Finally, we discuss the implications of SLU7 in pathophysiology, with particular emphasis on the progression of liver disease and its possible role as a therapeutic target in human cancer.

## 1. Introduction

**Figure 1:**
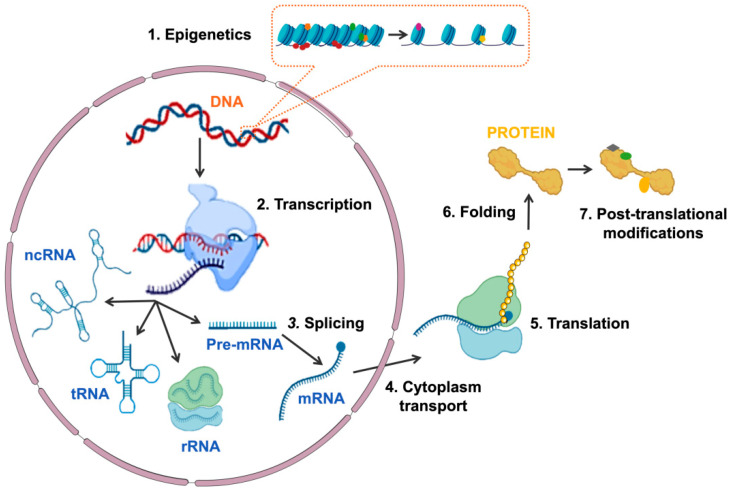
Schematic representation of the different mechanisms involved in regulating gene expression. The sequential regulatory events are: (1) epigenetics modifications at chromatin, (2) transcription of DNA to RNA, (3) splicing of pre-mRNA, (4) mRNA transport to cytoplasm, (5) translation of mRNA to protein, (6) protein folding and (7) protein post-translational modifications. Abbreviations: ncRNA, non-coding RNA; tRNA, transferring RNA; rRNA, ribosomal RNA; Pre-mRNA, pre-messenger RNA; mRNA, messenger RNA.

Gene expression is the process by which information encoded in DNA is transformed into functional products: either proteins or non-coding RNA (ncRNA) molecules. Gene expression is finely and dynamically controlled through the tightly coordinated and interconnected activity of multiple factors at different levels [1,2,3]. The first level of regulation is chromatin accessibility, which is determined by epigenetic marks: reversible modifications that, without altering gene sequence, determine chromatin structure and accessibility, leading to the activation or repression of transcription [4]. Transcription of both coding and non-coding genes begins with the recruitment of the RNA polymerase (RNA Pol) and the rest of the transcriptional machinery to the promoter region of the gene [5]. This is followed by the processing of the pre-messenger RNA (pre-mRNA) by splicing, which includes alternative splicing, so that different messenger RNAs (mRNAs) can be generated from the same pre-mRNA, widening the diversity of gene transcripts [6,7]. The mRNA is then directed to the cytoplasm, translated into an amino acid sequence in the ribosome and processed to produce a protein. Once the protein is generated, it can undergo a variety of post-translational modifications that determine its function, localization and half-life (Figure 1). The complexity and interconnection between the different levels of gene expression regulation are necessary to ensure the proper expression of genes in a cell-specific and temporal manner and, thus, guarantee cellular identity, function and viability [2].

The splicing factor synergistic lethal with U5 small nuclear RNA, SLU7, was first described in 1992 in yeast as an essential player in the second catalytic reaction of the splicing process [8]. Subsequent works identified its human homologue and characterized its role in the spliceosome [9,10,11,12,13,14], highlighting that SLU7 controls the diversity of gene transcripts in a cell. In addition, more recent findings have identified SLU7 also involved in other levels of gene expression regulation [15,16,17,18,19,20]. Specifically, SLU7 has been implicated in the epigenetic remodeling of DNA, in the modulation of the transcriptional activity and in controlling protein stability. All these findings identify SLU7 as a pleiotropic factor with a holistic function at different levels of gene expression regulation [15,16,17,18,19,20].

Here, we review the current knowledge on the role of SLU7 in gene expression regulation as well as its impact on relevant cellular functions. Moreover, we revise the implications of SLU7 in liver pathophysiology and its possible role as a therapeutic target in human cancer.

## 2. Characterization of SLU7

### 2.1. SLU7 Structure

**Figure 2:**
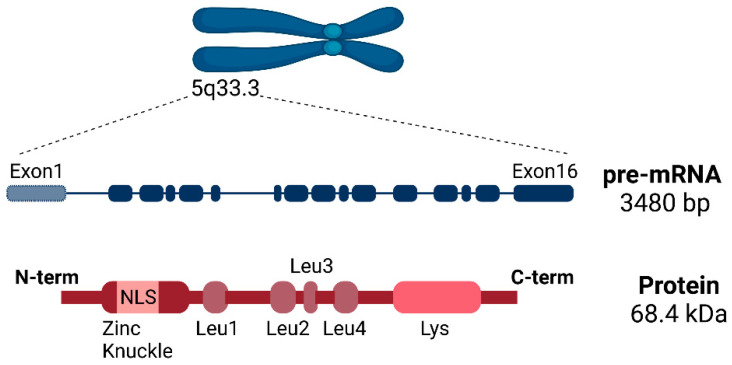
Schematic representation of human SLU7, depicting *SLU7*’s gene location on the long arm of chromosome 5 (5q33.3) and its pre-mRNA and protein structure. The pre-mRNA transcript contains 16 exons and is 3480 bp in length. The encoded protein weights approximately 68.4 kDa, and it contains four different domains: a nuclear location signal (NLS), a zinc knuckle motif, four stretches of leucine repeats and a lysine-rich domain.

The human *SLU7* gene is located on the long arm of chromosome 5 (5q33.3), where the antisense strand is transcribed into the *SLU7* mRNA composed of sixteen exons and originating from four alternative transcription start sites (TSS), not affecting the coding sequence, but changing the length of the 5′UTR [21]. Although different *SLU7* transcripts are described in both NCBI and Ensembl, to date, there is no information regarding their biological functions. The canonical *SLU7* transcript (NM_006425.5) is 3480 bp in length and encodes a protein of 586 amino acids (NP_006416.3) with an approximate molecular weight of 68.4 kDa [22]. Four motifs have been described in SLU7 protein that control its cellular localization and participate in its canonical function as a splicing factor: a nuclear location signal (NLS), a zinc knuckle motif, four stretches of leucine repeats and a lysine-rich domain [22] (Figure 2). The NLS targets SLU7 to the nucleus. The zinc knuckle motif, curiously embedded within the NLS, is not required for SLU7’s entrance into the nucleus but rather to maintain SLU7 inside it, preventing its shuttle back to the cytoplasm via the chromosomal region maintenance 1 (CRM1) pathway. In this regard, the four leucine repeats facilitate exporting SLU7 to the cytoplasm. In addition, the binding of zinc to the zinc knuckle motif and the lysine-rich region at the C-terminal domain also dictate the nuclear localization of SLU7 [22].

Cryo-electron microscopy studies revealed two large segments of SLU7, residues 62–196 at the N-terminal and residues 265–371 at the C-terminal, through which SLU7 interacts with PRP8 and CWC22 in the C* complex of the spliceosome [11]. Moreover, three regions at the N-terminal domain highly enriched in basic residues (Arg and Lys) are involved in the selection of 3′ splice sites (3′SS) during the second step of the splicing process [11].

Although not functionally characterized, there is evidence of several phosphorylation, acetylation, sumoylation and ubiquitylation sites in SLU7 protein (PhosphoSitePlus database [23]).

Strikingly, human SLU7 protein is 204 amino acids longer than its yeast homolog and shares an identity of only 24% in the zinc knuckle motif [9]. Likewise, this motif has high homology with the zinc knuckle motif of retroviral nucleocapsid proteins [24], implicated in retroviruses’ RNA packaging [25].

### 2.2. SLU7 Expression and Subcellular Localization

Similar to other splicing factors, SLU7 shows a broad expression in mouse and human tissues as well as in a wide variety of cell lines, although there exist differences in SLU7 mRNA and protein levels between cell types, developmental stages and aging [15,16,21,26,27]. For instance, stronger expression is detected in the brain, thyroid or testis, while lower expression is found in the pancreas or salivary glands [28]. Mouse *Slu7* transcripts are abundant in the developing heart [21], but negligible in the liver where its expression increases after birth [15,29].

The mechanisms that contribute to this different temporal and spatial expression of SLU7 are not well-characterized yet. At the transcriptional level, two transcription factors have been described to bind the *SLU7* promoter and regulate its expression in a dose-dependent manner: the specificity protein 1 (SP1) recognizes GC-rich promoter elements and promotes *SLU7* expression, while ETS-like gene 1 (ELK-1) recognizes Ets-binding sites (EBS) and inhibits *SLU7* transcription [21]. In this regard, the activation of signaling pathways may contribute to regulating *SLU7* expression, as demonstrated by its downregulation in transformed liver cells through the amphiregulin (AREG)/epidermal growth factor receptor (EGFR)/c-Jun NH2-terminal kinase (JNK)/ELK-1 pathway [30].

Interestingly, although both the mechanisms and the consequences are not completely defined, a physiological metabolic or nutritional regulation of SLU7 expression has been described in hepatic cells [15]. Therefore, in the mouse liver, SLU7 expression is higher in fasting rather than in refeeding conditions. Accordingly, at the transcriptional level, insulin inhibits *SLU7* expression in hepatic cells, and in pathological situations of hepatic insulin resistance, such as after long-term feeding with a high-fat diet, the expression of *SLU7* in the liver is induced [15].

Under physiological conditions, SLU7 is mainly accumulated in the nucleus, mostly with a speckled pattern [9,11,31]. Speckles are nuclear structures enriched in pre-mRNA splicing factors that generally co-localize with actively transcribed chromatin regions [32]. Accordingly, Western blot analyses after subcellular fractionation revealed an enrichment of SLU7 bound to chromatin [18,19]. As mentioned above, the NLS, the zinc knuckle motif and the lysine-rich domain of SLU7 dictate this predominantly nuclear localization [22].

Nevertheless, a shuttle of SLU7 protein to the cytoplasm has been described in a stress-dependent manner [31,33]. Exposure to stress stimuli in vitro, such as ultraviolet C irradiation or heat shock, triggers cytoplasmic SLU7 accumulation, subsequently altering the cell alternative splicing landscape [31]. Mechanistically, this nuclear-to-cytoplasm transport is dependent on the activation of JNK and, to lesser extent, of p38 (mitogen-activated protein kinase 14) cascades [31]. Similarly, thapsigargin-induced endoplasmic reticulum (ER) stress not only favors SLU7 cytoplasmic translocation but also promotes its distribution in vesicle-like organelles reminiscent of stress granules (SG) [33]. Accordingly, our in vivo studies revealed that acetaminophen (APAP)-induced acute liver damage also enhances cytoplasmic SLU7 translocation in hepatocytes, parallel to the activation of phospho-JNK [19]. Whether a specific phosphorylation of SLU7 and/or other post-translational modifications trigger stress-induced cytoplasmic transport remains to be elucidated, as well as the possible role of SLU7 in the stress-induced response. In fact, related to this point, we have recently shown that SLU7 binds in the cytoplasm to the well-characterized SG components Ras GTPase-activating protein-binding protein 1 (G3BP1) and ubiquitin-specific peptidase 10 (USP10) in unstressed cells and in normal mouse livers, consistent with the existence of pre-SG protein complexes that facilitate rapid SG formation under stress [19].

## 3. SLU7 Regulates Gene Expression at Different Levels

### 3.1. SLU7 as a Splicing Factor

#### 3.1.1. SLU7 and the Splicing Process

A common feature of eukaryotes is the presence of non-coding sequences, introns, interspersed between the coding regions of genes. Therefore, to form a mature mRNA which codes for a protein that is eventually synthesized in the cytoplasm, introns are removed and exons are ligated during the nuclear splicing reaction [34]. The splicing process takes place in two sequential transesterification reactions named branching and exon ligation [11,25,35,36]. In the first catalytic reaction, the spliceosome recognizes a guanine–uracil (GU) dinucleotide at the intron donor 5′ splice site and an adenosine residue within the branch point sequence, resulting in the cleavage of the 5′ exon from the intron. During the second reaction of exon ligation, the spliceosome recognizes an adenine–guanine (AG) dinucleotide at the acceptor 3′SS, triggering the cleavage of the intron at the 3′ exon border with concomitant ligation of the free 5′ exon and the 3′ exon [24,25,37]. After the catalysis, the spliceosome is disassembled, freeing the mature mRNA and the intron [37]. This complex reaction is driven by the spliceosome, a dynamic molecular complex composed of five types of small nuclear ribonucleoproteins (snRNPs) termed U1, U2, U5 and U4/U6 and a wide range of splicing factors which sequentially interact in coordination [14,38].

SLU7 was first isolated from yeast in 1992, when Frank and colleagues were screening for mutations that were synergistically lethal with loop mutations in the U5 snRNP and named those genes *SLU*—for synergistic lethal with U5 snRNP [8]. They identified four genes, *Slu1* and *Slu2*, essential for the first catalytic step of splicing, and *SLU7* and *SLU4*, which were required for the second step [8]. In particular, SLU7 was found to be essential for the correct selection of the 3′SS during the second step of the splicing process [24]. Moreover, it was described that the requirement of SLU7 for this selection is not based on the recognition of a specific sequence, but rather dictated by the distance between the branch point and the AG dinucleotide. In this regard, when the distance between the 3′SS and the branch point is shorter than 7 nucleotides, the presence of SLU7 is not necessary [39]. However, with greater distances, SLU7 is mandatory to provide efficient docking of the 3′SS to the active site [39,40,41].

In 1998, Chua and Reed isolated the human SLU7 homolog, hSLU7, and demonstrated that, as in yeast, this splicing factor is essential for the correct selection of the AG 3′SS during the second catalytic reaction [9]. Importantly, the lack of hSLU7 results in a random attack on AG dinucleotides independent of its position, the accumulation of products from the splicing step and the stalling of splicing between the catalytic steps [9]. Mechanistically, the role of SLU7 in the selection of the 3′SS could be explained by its unusually high content of positively charged amino acids, which allow SLU7 to bind the negatively charged pre-mRNA sequences of the 3′ intron lariat [11].

In recent years, cryo-electron microscopy assays have described the atomic structure of the different complexes forming part of the human spliceosome [11,12,13]. Thus, the fully assembled spliceosome exists in eight major functional states: precursor of the pre-catalytic spliceosome (pre-B), pre-catalytic spliceosome (B), activated spliceosome (Bact), catalytically activated spliceosome (B*), catalytic step I complex (C), step II catalytically activated complex (C*), post-catalytic spliceosome (P) and intron lariat spliceosome (ILS). Branching and exon ligation occur in the B* and C* complexes, respectively [12]. SLU7 is specifically recruited to the spliceosome machinery in the transition of pre-C*-I to pre-C*-II intermediate states [13]. In particular, SLU7 interacts with PRP8 and CWC22 and it is placed close to the spliceosome’s active site, favoring its orientation towards the 3′SS for the second catalytic step [11,14,38]. After this second reaction, the C* complex is rearranged into the post-catalytic spliceosome complex (P) in order to stabilize the 3′SS into the active site. In this complex, the SLU7 N-terminal domain is located close to the spliceosome active site to facilitate the transfer of the 3′SS to the active site. This key location also allows its binding to PRP8 to stabilize its conformation [12]. In the P complex, the ligated exon is locked in the catalytic center formed by PRP8 and the RNA elements, and the role of SLU7 is to protect these interactions from being broken early. It is not until the PRP22 signal in the P-to-ILS transition that the ligated exon is released and the protein components, including SLU7, are disassembled [12].

#### 3.1.2. Examples of Splicing Events Associated with SLU7 Activity

Alternative splicing is cell-specific and responds to environmental signals [42]. Therefore, the relative proportion of the different isoforms determines cellular identity and adaptation and varies during development, aging, physiological responses and diseased states [26,29,43]. Consequently, the dysregulation of constitutive and alternative precursor mRNA splicing processes is implicated in multiple human diseases, including cancer [37,44]. Thus, in tumors, splice site mutations or impaired expression and/or activity of splicing factors participate in the oncogenesis process, altering the dotation of mRNA isoforms [45,46].

**Figure 3:**
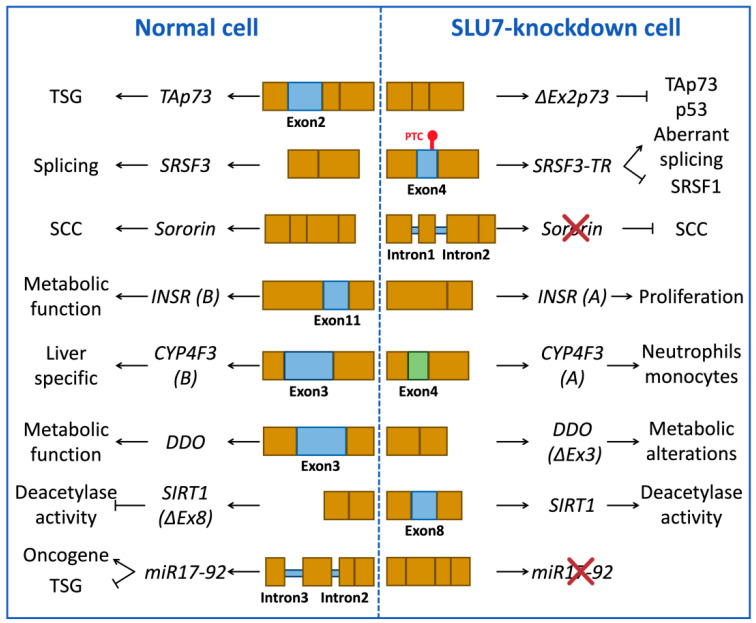
Splicing events in normal and SLU7-knockdown cells. Examples of constitutive and aberrant splice variants in normal and SLU7-knockdown cells and their cellular consequences. Some examples are the tumor suppressor gene *TAp73*, the splicing factor *SRSF3*, the gene implicated in sister chromatid cohesion (SCC) *sororin*, the metabolic insulin receptor (*INSR*), the metabolic enzymes cytochrome P450 (*CYP4F3*) and D-aspartate oxidase (*DDO*), the deacetylase enzyme sirtuin 1 (*SIRT1*) and the polycistronic microRNA cluster *miR17-92*. Blue or green boxes represent the alternative exons or introns. Abbreviations: PTC, premature termination codon; SCC, sister chromatid cohesion; TSG, tumor suppressor gene; SRSF3-TR, truncated SRSF3.

The impact of SLU7 in the composition of the whole transcriptome depends on the cell type and the cellular state. We herein gather some examples of reported endogenous splicing events controlled by SLU7 both in physiological and pathological conditions including transcription factors such as *TP73* [30], splicing factors such as serine/arginine-rich splicing factor 1 and 3 (*SRSF1* and *SRSF3*) [15,17], microRNAs such as the cluster *miR17-92* [16], metabolic receptors such as the insulin receptor (*INSR*) [15], metabolic enzymes such as the cytochrome P450 family 4 subfamily F member 3 (*CYP4F3*) [15], D-aspartate oxidase (*DDO*) [31] and NAD+-dependent class III deacetylase sirtuin 1 (*SIRT1*) [20] and genes implicated in mitotic chromosome segregation such as *sororin* [17] (Figure 3).

Multiple isoforms with different biological activities have been described for the transcription factor tumor suppressor gene *TP73* [47,48,49]. Among them, p73 variants lacking the N-terminal transactivation domain (TA), named DeltaTAp73, can be generated by both alternative promotor usage or alternative splicing of exons 2, 3, or 2 and 3 together [47,48]. These truncated p73 isoforms are overexpressed in several pre-neoplastic conditions including liver cirrhosis [30] and in different types of cancer, playing oncogenic roles as they act as dominant negative inhibitors of both TAp73 and the tumor suppressor p53 [47,50]. SLU7 favors exon 2 inclusion and the correct splicing of *p73* into the tumor suppressor full-length isoform *TAp73* [30]. Thus, the downregulation of SLU7 levels described during chronic liver disease and hepatocellular carcinoma (HCC) explains, in part, the accumulation of *DeltaTAp73* transcripts in these pathologic conditions [30], which could contribute to carcinogenesis as demonstrated in mice [51].

Serine- and arginine-rich (SR) proteins are RNA-binding proteins (RBPs) known as constitutive and alternative splicing regulators [52] that are also subjected to alternative splicing. The pre-mRNA alternative splicing of *SRSF3* can generate the full-length isoform 1 lacking exon 4 (Iso1) and the alternative isoform 2 including exon 4 (Iso2), which contains an aberrant premature termination codon (PTC) promoting SR transcript degradation or, as we have demonstrated, the generation of truncated protein isoforms [53]. SLU7 regulates *SRSF3* splicing [15,17,20], although contradictory results have been published. On one hand, we have described that SLU7 is required for exon 4 exclusion and the generation of the *SRSF3* Iso1 transcript both in vitro and in vivo [15]. Thus, SLU7 knockdown triggers an increase in *SRSF3* isoform 2 containing the PTC [15], which would code for truncated SRSF3 proteins (SRSF3-TR) [17]. These truncated proteins could behave as dominant negative species toward SRSF3 activity and/or have a specific function being, for instance, responsible for the aberrant splicing and expression of the SR protein SRSF1 or of sororin, an essential protein in sister chromatid cohesion (SCC) [17], as discussed below, participating in the process of hepatocarcinogenesis [17]. On the other hand, it has been also published that adenovirus-mediated knockdown of SLU7 prevented an increase in the *Srsf3*-Iso2/Iso1 ratio induced in ethanol-fed mice [20]. Further experiments are required to elucidate whether these discrepancies are due, for instance, to the hepatic-specific [17] or ubiquitous [20] modulation of SLU7 expression in vivo. In any case, the findings demonstrate that SLU7 plays a relevant role in controlling the splicing of very prominent splicing factors, thus amplifying its relevance in maintaining this essential process.

The INSR has two splice variants [54,55]: the isoform B, which includes exon 11, and the isoform A, which excludes exon 11 [56]. INSR-B is highly expressed in pancreatic beta cells, the liver and also in muscle, adipose tissue and the kidney; it is involved in metabolic signaling, whereas INSR-A is predominantly expressed in fetal development and in certain cancers, being involved in cell proliferation [54,57]. The correct expression of SLU7 is required to generate the *INSR*-B splice variant, as liver-specific SLU7-knockdown cells induced an increase in the *Insr*-A/B ratio [15].

The cytochrome P450 4F3 (CYP4F3) participates in the metabolism of various endobiotics, as well as some xenobiotics, and it has two isoforms: the original CYP4F3A isoform detected in neutrophils and monocytes, and CYP4F3B expressed in fetal and adult liver [58,59,60]. Alternative splicing of mutually exclusive exons 3 and 4 generates these isoforms. *CYP4F3A* contains exon 4, whereas *CYP4F3B* contains exon 3 [58,59]. SLU7 is necessary in the liver to ensure the selection of exon 3 and, thus, generate the hepatic isoform *CYP4F3B* [15]. Regarding other metabolic genes, a reduction in SLU7 nuclear concentration by stress (ultraviolet irradiation) or RNA interference treatment affects the alternative splicing of the *DDO* gene, which is implicated in glyoxylate metabolism and glycine degradation, from mostly exon 3 inclusion to mostly exon 3 skipping, encoding an insoluble protein whose functional role could not be determined [31]. Another example includes SIRT1 in mouse hepatocytes. Adenovirus-mediated SLU7 expression induces the skipping of SIRT1 exon 8. Consequently, knockdown of hepatic SLU7 prevents the induction of SIRT1-DeltaExon 8 after ethanol administration in mice [20].

The polycistronic microRNA cluster *miR-17-92* is considered oncogenic and it is overexpressed in a wide range of malignancies including hematological and solid tumors such as those found in the breast, colon, lung and liver [61,62,63,64]. The *miR-17-92* cluster is located in the third intron of a primary transcript known as *C13orf25* and is expressed when *C13orf25* transcript B containing introns 2 and 3 is generated [64,65]. Our RNA-crosslinking immunoprecipitation (RNA-CLIP) study in cancer cells revealed that SLU7 directly binds *C13orf25* mRNA to ensure the expression of transcript B and the generation of the miRNAs implicated in cancer cell survival [16].

Sister chromatids remain physically connected from the moment of their synthesis during DNA replication until their dissociation during anaphase in mitosis. This sister chromatid cohesion (SCC) is essential for biorientation of chromosomes on the spindle in the metaphase plate for a correct chromosome segregation [66,67]. Sororin is an essential protein for SCC [68,69]. SLU7-knockdown cells displayed defects in SCC, with a failure of chromosomes to congress at the metaphase plate, as well as defects in spindle polarity [17]. Mechanistically, SLU7 controls the expression of *sororin* through its correct pre-mRNA processing. Thus, SLU7 knockdown results in the accumulation of unspliced *sororin* with increased retention of introns 1 and 2, generation of PTC-containing isoforms and reduced levels of sororin protein [17]. Importantly, this aberrant intron retention and reduced sororin expression, associated with a defect in mitosis progression, was also validated in vivo when SLU7 was silenced (AAV-shSLU7) in hepatocytes in the context of liver regeneration after partial hepatectomy [17]. Mechanistically, the aberrant splicing of *sororin* was associated with the reduced expression of *miR-17* and the accumulation of SRSF3-TR proteins upon SLU7 knockdown [17].

### 3.2. SLU7 as a Transcription Regulator

**Figure 4:**
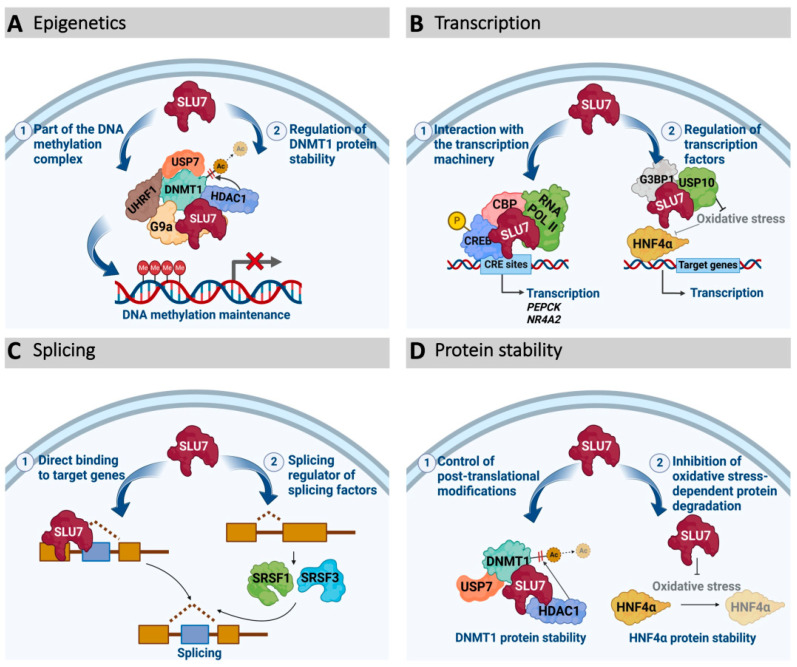
Schematic representation of SLU7 participation at different levels of gene expression regulation. (**A**) SLU7 acts as an epigenetic modulator by participating in the DNA methylation complex and by regulating the protein stability of DNMT1. (**B**) SLU7 regulates transcription by interaction with transcriptional complexes (cAMP-induced complex) or by regulating the expression of transcription factors (HNF4α1). (**C**) SLU7, through its canonical function as a splicing factor, regulates the splicing of numerous genes, either by direct binding to different targets or by regulating the splicing and expression of other splicing factors. (**D**) SLU7 regulates protein stability through the control of post-translational modifications, as in the case of the acetylation of DNMT1, or by inhibiting oxidative stress-mediated protein degradation, as in the case of HNF4α1.

The transcriptomic program is regulated by the recruitment of the RNA Pol and the rest of the transcriptional machinery to the promoter region of genes in response to intra- or extra-cellular signals. Our results demonstrate that SLU7 regulates transcription by at least two different mechanisms (Figure 4B), as part of transcriptional complexes [15] and regulating the stability of transcription factors [19].

The cyclic adenosine 3′,5′-monophosphate (cAMP) signaling pathway is a pivotal regulator of metabolism, cell proliferation, differentiation and apoptosis in endocrine tissues [70] including the liver [71]. cAMP activates gene transcription by phosphorylating CRE-binding protein (P-CREB, cAMP response element-binding protein) and promoting its association with coactivators such as CREB-binding protein (CBP), which recruits the RNA Pol II on cAMP response elements (CRE) containing promoters [72]. In this sense, our group has demonstrated that SLU7 interacts with the RNA Pol II and protein members of the cAMP-responsive pathway such as P-CREB and CBP [15], thus regulating the expression of cAMP-dependent gluconeogenic genes such as phosphoenolpyruvate carboxykinase (*Pepck*) or nuclear receptor subfamily 4 group A member 2 (*Nr4a2*), suggesting a direct role of SLU7 in regulating transcription.

On the other hand, we have recently found that SLU7 is required to protect the transcription factor hepatocyte nuclear factor 4 alpha 1 (HNF4α1) from its oxidative stress- and PKC (protein kinase C)-mediated degradation [19]. More specifically, in hepatic cells, SLU7 is required to maintain the expression and, therefore, the antioxidant activity of the ubiquitin-specific peptidase 10 (USP10), stabilizing HNF4α, its transcriptional program and the correct usage of HNF4α P1 and P2 promoters [19,73,74].

### 3.3. SLU7 as an Epigenetic Modulator

Epigenetics refers to reversible modifications that do not alter gene sequence but determine chromatin structure and its accessibility, resulting in transcription activation or repression [4,75]. Among them, DNA methylation consists of the addition of methyl groups to the 5′ position of cytosine residues on CpG dinucleotides [76]. These CpG dinucleotides are distributed throughout the genome, but are enriched in regions called CpG islands (CGIs) that are generally located at promoter regions where, when methylated, are associated with gene repression [4]. On the contrary, the methylation of CGIs located in the gene body has been associated with transcription activation [77,78]. DNA methylation patterns are faithfully inherited from the mother DNA molecule to the daughters after each cell division [79,80]. DNA methyltransferase 1 (DNMT1) is the enzyme responsible for this DNA methylation maintenance [79,80,81,82].

Surprisingly, we found that SLU7 regulates DNA methylation (Figure 4A) and, therefore, SLU7 silencing in a wide variety of cancer cells, resulting in a general DNA hypomethylation [18]. Mechanistically, we showed that SLU7 is required to secure DNMT1 protein stability and, therefore, a correct DNA methylation inheritance in proliferating cells. Our results demonstrate that SLU7 interacts with DNMT1, being required for the binding of the histone deacetylase 1 (HDAC1) to DNMT1, preventing DNMT1 acetylation and securing DNMT1 interaction with the deubiquitinating enzyme ubiquitin-specific peptidase 7 (USP7) to stabilize DNMT1 protein. Importantly, SLU7 not only interacts and regulates DNMT1 stability, but it also interacts in the chromatin with different members of the protein complex implicated in DNA methylation maintenance such as the DNMT1 adaptor protein UHRF1 (ubiquitin-like with PHD and ring finger domains 1) and the histone methyltransferase G9a (euchromatic histone lysine methyltransferase 2), regulating their integrity [18]. Therefore, SLU7 knockdown resulted in DNMT1, UHRF1 and G9a decay, a general hypomethylation of DNA, and the induction of expression of genes normally silenced by promoter methylation, including tumor suppressor genes, endogenous retroviruses and cancer-testis antigens (CTAs) [18].

### 3.4. SLU7 as a Protein Stability Regulator

As described above, the functions of SLU7 at different levels of gene expression regulation are, in many cases, associated with an unexpected role of SLU7 governing the stability of different proteins (Figure 4D). As already mentioned, SLU7 secures DNMT1 protein stability, preventing its acetylation, subsequent ubiquitination and proteasomal degradation, mediating the binding of DNMT1 to HDAC1 [18]. Moreover, SLU7 is required to maintain the SG antioxidant components G3BP1 and USP10, as SLU7-knockdown cells reduce their protein levels without affecting their mRNAs [19]. Importantly, the modulation of USP10 expression and the induction of oxidative stress upon SLU7 knockdown, in turn, leads to the loss of HNF4α1 protein expression [19] (Figure 4D).

## 4. SLU7 Functions

As mentioned above, SLU7 regulates DNA methylation, gene transcription, mRNA splicing and protein stability, affecting the expression of many proteins with multiple functions. Experiments modulating SLU7 expression have demonstrated a requirement to preserve relevant functions including cell differentiation, genome stability, antioxidant responses and cell cycle progression. Therefore, although very little is known about the mechanisms controlling SLU7 expression, cells require a precise control of both the level of SLU7 expression and its localization [15].

### 4.1. SLU7 and Liver Differentiation

A few years ago, we demonstrated that SLU7 is essential to maintain the differentiation, the metabolic function and the quiescence of hepatocytes [15,29] through the different mechanisms already detailed above. Accordingly, in vivo *SLU7* knockdown in mouse and in human liver cells resulted in profound changes in pre-mRNA splicing and gene expression, promoting hepatocellular de-differentiation and impairing metabolic responses [15]. Importantly two key regulators of hepatocellular differentiation and function, SRSF3 [83] and HNF4α1 [84], are regulated by SLU7 [15,17,19]. Hepatic *Slu7* knockdown [15] or haploinsufficiency [19] decreases the expression of both SRSF3 and HNF4α1 and other essential transcription factors such as *Hnf1α* (hepatocytes nuclear factor 1 α), liver characteristic genes such as albumin and genes involved in lipid, cholesterol or glucose metabolism, such as the enzymes fatty acid synthase (*Fasn*), sterol regulatory element-binding proteins (*Srebp*), glycogen synthase 2 (*Gys2*), PEPCK or glucose-6-phosphatase (*G6PC*) [15]. In addition, reduced levels of SLU7 also induce the expression of fetal markers such as alpha fetoprotein (*Afp*), *H19*, *Mat2a* (methionine adenosyltransferase 2A) or *Wt1* (Wilms’ tumor 1), and fetal-like isoforms or genes (for instance, *InsrB* and *Cyp4f3B*) [15]. Moreover, SLU7 downregulation also induces stress-response genes such as activating transcription factor 3 (*Atf3*) or *Tp53*, increases hepatocellular proliferation and induces a metabolic switch to a tumor-like glycolytic phenotype, triggering the expression of hexokinase 2 (*Hk2*) and pyruvate kinase m2 (*Pkm2*) and reducing the corresponding isoforms glucokinase (*Gck*) and L-pyruvate kinase (*Lpk*) [15]. Taken together, these observations highlight the existence of a hierarchical and complex network of gene expression regulation involved in the maintenance of the adult liver phenotype, in which SLU7 plays a major role [29].

### 4.2. SLU7 and Cellular Response to Stress

In both cultured cells and mouse liver cells, SLU7 knockdown induces oxidative stress [16,19]. Several gene expression alterations could be related to an increase in ROS (reactive oxygen species), including the downregulation of antioxidant enzymes targets of HNF4α1 such as SOD2 (superoxide dismutase 2) [19,85] or the impaired processing of miR17-92 [16,86]. Moreover, recent results demonstrate that SLU7 is required to secure the formation of SG, an essential part of the cell’s antioxidant machinery [19]. SGs are assemblies of translating messenger ribonucleoproteins (mRNPs) formed to protect mRNAs upon stress-mediated translation inhibition [87,88]. Interactions between stress granule proteins exist ahead of a stress response [89,90], facilitating the formation of SG upon stress. In fact, SLU7 interacts with several SG core components such as G3BP1 and USP10 [19], both with antioxidant activity [89,91,92], in unstressed cells [19]. Moreover, SLU7 is required to maintain the levels of expression of G3BP1 and USP10 and, therefore, upon SLU7 knockdown, the number of SG granules formed in response to stress is reduced [19].

The participation of SLU7 in the cellular response to stress could be also associated with its different localization. As already mentioned, it has been found that stress conditions alter the cellular distribution of SLU7, inducing its cytoplasmic shift [33]. Importantly, in vivo, this has been demonstrated in response to the induction of an acute damage to hepatocytes upon APAP treatment [19]. This suggests that the differential distribution of SLU7 upon stress might be a regulatory mechanism by which SLU7’s nuclear protein levels are regulated [31] and cytoplasmic functions are enhanced.

### 4.3. SLU7 and Genome Stability

The preservation of genome integrity is essential for cellular homeostasis and survival. DNA lesions, and the failure to repair them, can lead to the accumulation of mutations [93]. Moreover, mitotic alterations can lead to chromosome aberrations and aneuploidy [94]. An increased rate of these events is termed genome instability, and it has been associated with neoplastic diseases, representing one of the hallmarks of cancer [95,96].

Again, experiments performed with normal mouse liver cells as well as in a wide variety of cancer cells of different origins demonstrate that SLU7 is a core factor in the preservation of genome integrity acting at different levels [17]. As mentioned before, SLU7 prevents the accumulation of ROS [16,19], which are important players in the induction of DNA damage [97]. Consequently, SLU7 knockdown results in an accumulation of γH2AX both in vitro and in vivo [16,19]. Moreover, SLU7 prevents the induction of transcription-associated genome instability (TAGIN) [17]. Although a direct role of SLU7 in protecting the nascent mRNA from the formation of RNA–DNA hybrids (R-loop) cannot be discarded, SLU7 mediates the splicing of *SRSF3* [15], favoring SRSF3 Iso1 isoforms that, in turn, ensure the correct splicing and expression of SRSF1 [98,99], which has been associated with protection against the formation of transcription-associated R-loops and TAGIN [94]. Lastly, SLU7 is required to prevent the accumulation of mitotic alterations by securing the correct expression of SRSF1 and a bipolar spindle formation [100] and by controlling the splicing and correct expression of sororin and the correct cohesion of sister chromatids at the metaphase plate [17].

All these data underscore the importance of SLU7 in ensuring cellular homeostasis and viability, as well as preserving fidelity in the transmission of genetic information between cell generations. Importantly, R-loops are known to interfere with DNA replication and to induce replication stress, which has been proposed as an exploitable therapeutic vulnerability of cancer cells, as is discussed below [101,102].

### 4.4. SLU7 and the Cell Cycle

As already mentioned, the role of SLU7 in the cell cycle seems to be dual. On one side, SLU7 is necessary to ensure the quiescence of the hepatocytes [15]; however, it is also required to secure a correct progression through the cell cycle during liver regeneration and, in general, in transformed proliferating cells [17].

Indeed, SLU7 knockdown in the liver induces the entry of the hepatocytes into the cell cycle [15]. Although the mechanism has not been elucidated, transcriptome rewiring may contribute to this effect. In fact, the induction of *HK2* and *PKM2* isozymes, which have been related to the induction of proliferation and transformation [95,103] as well as the upregulation of *Foxm1* (forkhead box M1), *Nor1* (neuron-derived orphan receptor-1), *Egr1* (early growth response 1) and the cyclins *Ccnd1*, *Ccna2* and *Ccnb2*, will enable the entry of the hepatocytes into the cell cycle in response to a partial hepatectomy (PH) [15]. In fact, in hepatocytes, during liver regeneration after PH, there is an early and temporary decrease in SLU7 expression to allow for the entry of quiescent hepatocytes into the cell cycle [15]. However, in line with previous studies showing that spliceosomal components play a direct role in cell division, and that their depletion induces the arrest at different phases of the cell cycle [104,105,106], SLU7 is also essential to ensure cell cycle progression [17]. Therefore, after the decay in the initial phases of the PH, SLU7 expression levels need to be recovered in the regenerating and cycling hepatocytes to ensure the proper progression of the cell cycle and, finally, to allow the restoration of the liver mass [17].

Importantly, SLU7 is also essential to ensure cell cycle progression of cancer cells of different origins [17]. Mechanistically, a reduction in SLU7 through the inhibition of the *miR-17-92* cluster induces the expression of P21 (cyclin-dependent kinase inhibitor 1A) [16], an important cell cycle inhibitor [107]. Accordingly, *miR-17* expression prevents cell cycle arrest and restores cell cycle progression in transformed SLU7-knockdown cells [17]. Moreover, SLU7 knockdown impairs spindle assembly and sister chromatid cohesion, at least in part, through the aberrant expression of sororin and SRSF1, promoting the accumulation of mitotic aberrations, which altogether ultimately leads to mitotic arrest and cell death [17]. This dependency of tumor cells on SLU7 levels to keep cycling, proliferating and surviving [16] could be also associated with its role in maintaining DNMT1 levels and a correct DNA methylation, as DNMT1 silencing induces G2/M arrest and apoptosis [108]. All in all, SLU7 has a central role in regulating cell proliferation and survival, positioning SLU7, as discussed below, as a promising antitumor therapeutic target.

## 5. SLU7 in Human Disease

### 5.1. SLU7 Expression in Pathological Conditions

Preliminary evidence of changes in SLU7 levels in a pathological condition was in the context of human inflammatory bowel disease [109]. *SLU7* was found downregulated in the colonic mucosa of patients with ulcerative colitis independent of the inflammation status of the tissue. Remarkably, in the mucosa of patients with Crohn’s disease, *SLU7* was downregulated in inflamed tissues but not in non-inflamed tissues [109]. Therefore, the different expression patterns of *SLU7* in these two inflammatory bowel diseases pointed to SLU7 as a potential discriminator between ulcerative colitis and Crohn’s disease [109]. Additionally, genome-wide association studies (GWAS) identified SLU7 single-nucleotide polymorphisms (SNPs) associated with tobacco smoke risk for inflammatory bowel disease (rs41275313) [110].

Evidence demonstrates alterations in SLU7 expression in the liver during the development of liver injury. A significant reduction at the mRNA level [30], that has been recently confirmed at the protein level [19], was observed during the progression of human liver disease from cirrhosis to HCC as well as in mouse models of acute and chronic liver damage [19,30]. This downregulation was associated with the activation of ELK-1 upon binding of AREG to the EGFR and the sequential activation of JNK-1 [30]. Therefore, the induction of AREG expression in the liver in response to inflammatory signals [111,112] would explain the [96] downregulation of SLU7 after liver injury or during the process of hepatocarcinogenesis [15,19,113]. In contrast, hepatic SLU7 was reported to be upregulated in ethanol-fed mice and in patients with alcoholic steatohepatitis, although the underlying mechanism was not described [20]. The induction of SLU7 was also observed in the liver of mice fed a high-fat diet, which could be explained by the presence of insulin resistance, as insulin inhibits SLU7 expression during the physiological cycle of fasting and refeeding [15]. All in all, further studies are needed to clarify the regulation of SLU7 expression in the damaged liver.

Although no link between SLU7 and hematopoiesis has been made so far, SLU7 is located in the region of the long arm of chromosome 5 (from 5q14.1 to 5q35.1) frequently deleted in myelodysplastic syndromes (MDS) [114]; therefore, SLU7 downregulation would be expected in MDS. Additionally, SLU7 has been recently identified as a susceptible locus for systemic lupus erythematosus (rs1895321) [115]. Although these findings highlight the presence of alterations in SLU7 levels during various pathological processes, the implications of these changes in expression are, in most cases, unknown. Therefore, further experiments and studies are needed to find out whether SLU7 changes are implicated in other pathologies, to characterize the mechanisms governing SLU7 expression and to elucidate the role of SLU7 in disease, including liver disease, inflammatory bowel disease and myelodysplastic syndromes. Next, we review the current knowledge of the implications of SLU7 in the context of liver diseases.

### 5.2. SLU7 in Liver Pathology

**Figure 5:**
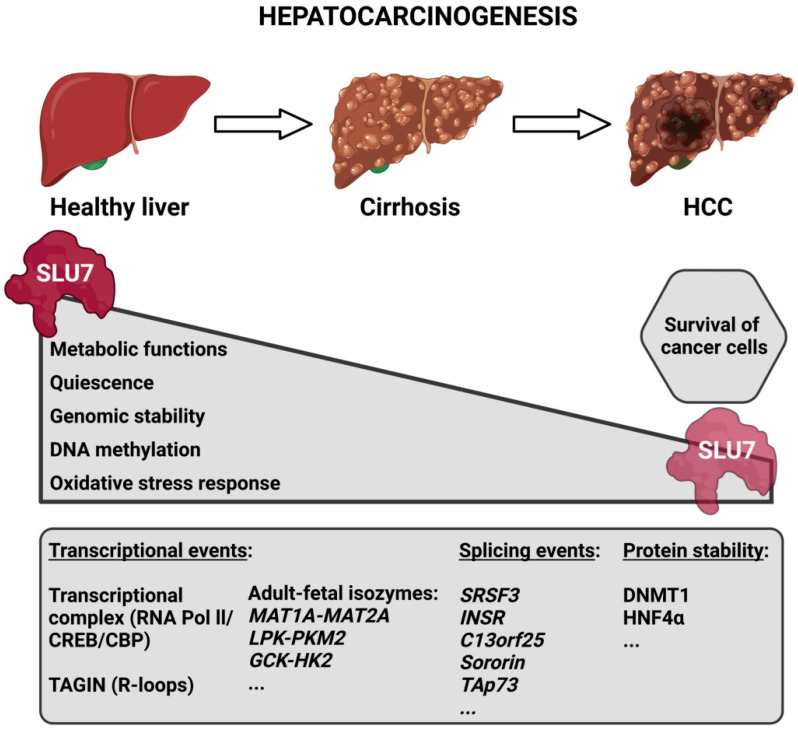
Schematic representation depicting SLU7’s involvement in the process of hepatocarcinogenesis. During the progression of liver disease, the expression of SLU7 decreases in parallel with the alteration of essential cellular functions. However, the residual SLU7 expression in tumoral cells is essential for their survival. The bottom box depicts some SLU7-controlled gene expression events that are altered during liver disease progression. Abbreviations: HCC, hepatocellular carcinoma; TAGIN, transcription-associated genome instability.

Given the role of SLU7 in the maintenance of hepatocellular identity [15], it is reasonable to speculate that the decreased expression of SLU7 observed during the progression of liver disease [19,30] could be involved in the de-differentiation of the hepatocytes and the loss of liver functions observed in patients, which is indicative of their bad prognosis. In fact, our results have demonstrated that *Slu7* haploinsufficiency in mice is enough not only to exacerbate liver dysfunction and potentiate transcriptome rewiring, but also to sensitize the liver to both acute and chronic damage [19]. In fact, all the data concerning the different functions of SLU7 described above suggest that the reduction in SLU7 levels observed in the livers of patients [19,30] contributes to the process of hepatocarcinogenesis. Indeed, it contributes to the reactivation of oncofetal isoforms and proliferation genes [15,116], to the generation of oxidative stress and DNA damage [16], to the induction of genomic instability, both through aneuploidy and mutations [17,117], and to the general DNA hypomethylation [18,118] observed in patients during the process of hepatocarcinogenesis (Figure 5).

Moreover, it is important to note that our results demonstrate that preventing SLU7 downregulation in damaged mouse liver cells helps to preserve the hepatocellular functions restoring hepatocellular differentiation and, importantly, protects the liver against chronic injury [19]. Therefore, these results reinforce the potential of differentiation therapies such as, for instance, HNF4α1 [119,120] or CBPα [121,122] restitution to treat liver diseases [29], presenting SLU7 as a new target.

### 5.3. SLU7 in Cancer

Based on the demonstrated functions of SLU7, it is clear that SLU7 is required to preserve cellular homeostasis and genomic integrity. Accordingly, any situation favoring its downregulation could contribute to the process of carcinogenesis, as has been suggested in the liver. However, it is important to highlight that, perhaps due to these central and pleiotropic roles linked to cell proliferation, SLU7 is a non-redundant survival factor for human cancer cells of very different origins and does not affect the viability of normal cells [16]. Thus, in this proliferative context, SLU7 could help to secure cell cycle progression [16] and DNA methylation maintenance [18], the expression of survival oncogenes such as the microRNA cluster miR17-92 [16], and to limit the mitotic stress associated to R-loop accumulation [17].

Therefore, SLU7 could represent a cancer cell’s vulnerability, and the silencing of SLU7 could represent a new therapeutic strategy [16]. In fact, SLU7 knockdown could recapitulate many events that have been proposed separately as anti-cancer strategies, including global DNA demethylation [123,124], induction of R-loop formation [101,102,125,126], DNA damage [101,102], cell cycle arrest [127,128], mitotic stress [101,102], autophagy [129,130] and apoptosis [131,132]. Future studies should help to elucidate whether, as suggested, SLU7 represents a pan-cancer therapeutic target.

## 6. Conclusions

**Figure 6:**
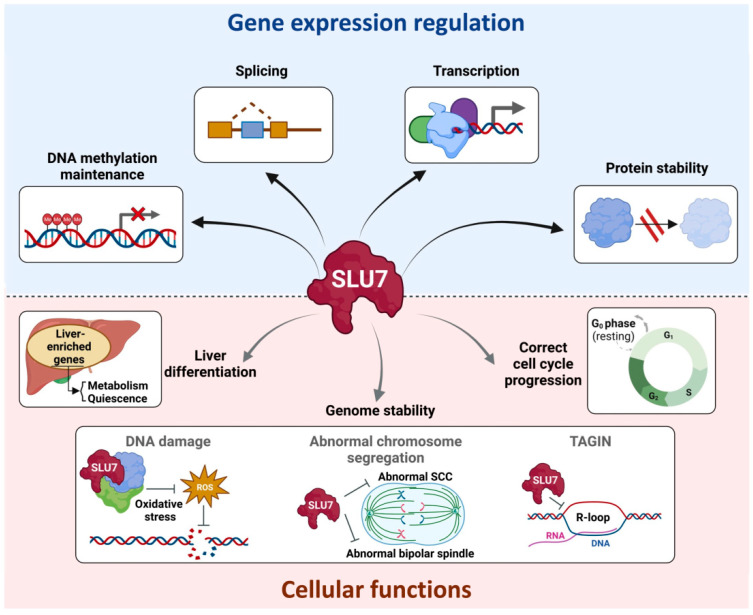
Schematic representation compiling the involvement of SLU7 in the regulation of gene expression at different levels and in cellular functions. See text for details. Abbreviations: ROS, reactive oxygen species; SCC, sister chromatid cohesion; TAGIN, Transcription-associated genome instability.

Although SLU7 is still poorly explored, knowledge to date highlights SLU7 as an integrative hub of different levels of gene expression regulation, including its canonical and essential function as an mRNA splicing factor, but also new and non-redundant roles in epigenetic DNA remodeling, modulation of promoter activity and protein stability (Figure 4). Through this widespread gene expression regulation, SLU7 is essential for multiple biological functions such as ensuring the identity and correct function of the liver, genome stability, cell cycle progression and stress response (Figure 6). Thus, altered expression of SLU7 could contribute to the progression of different diseases. Moreover, SLU7 could represent a cancer cell’s vulnerability to be therapeutically explored. Further research is needed to better understand SLU7 regulation and function and to define therapeutic avenues wherein regulating SLU7 levels or SLU7 functions could have clinical relevance.
